# Chemically Dual-Modified Biochar for the Effective Removal of Cr(VI) in Solution

**DOI:** 10.3390/polym14010039

**Published:** 2021-12-23

**Authors:** Juanjuan Yang, Yu Song, Yan Yue, Wenfei Liu, Quande Che, Honglei Chen, Hongfang Ma

**Affiliations:** 1 State Key Laboratory of Biobased Material and Green Papermaking, Qilu University of Technology (Shandong Academy of Sciences), Jinan 250353, China; m13678817612@163.com (J.Y.); 1043119575@stu.qlu.edu.cn (Y.S.); yueyan@qlu.edu.cn (Y.Y.); chenhonglei_1982@163.com (H.C.); 2 School of Environmental Science and Engineering, Qilu University of Technology (Shandong Academy of Sciences), Jinan 250353, China; 3 Department of Chemistry and Biochemistry, University of California, Los Angeles, Los Angeles, CA 90095, USA; wenfei95@gmail.com; 4 School of Materials Science and Engineering, University of Jinan, Jinan 250022, China; mse_cheqd@ujn.edu.cn

**Keywords:** biochar, Al/Mn modification, Cr(VI) absorption, removal mechanism, wastewater treatment

## Abstract

Here, a dual-modification strategy using KMnO_4_ (potassium permanganate) and AlCl_3_·6H_2_O (aluminum chloride, hexahydrate) as co-modifiers to improve the Cr(VI) removal capacity of K_2_CO_3_ activated biochar is introduced. As a result, the dual-modified biochar with KMnO_4_ and AlCl_3_·6H_2_O has the calculated adsorption energy of −0.52 eV and −1.64 eV for HCrO_4_^−^, and −0.21 eV and −2.01 eV for Cr_2_O_7_^2−^. The Al_2_O_3_ (aluminum oxide) and MnO (manganese oxide) embedded on the surface of dual-modified biochar bring more Cr(VI) absorption sites comparing to single-modified biochar, resulting in a maximum Cr(VI) saturated adsorption capacity of 152.86 mg g^−^^1^. The excellent removal performance is due to the synthetic effect of electrostatic attraction, reduction reaction, complexation reaction, and physical adsorption. The experimental results also indicated that the spontaneous adsorption process agreed well with the pseudo-second order and Langmuir models. This dual-modification strategy is not limited to the treatment of Cr(VI) with biochar, and may also be incorporated with the treatment of other heavy metals in aqueous environment.

## 1. Introduction

Discharge of chromium-containing wastewater has led to destruction of the aquatic ecological environment. Among all the oxidation states of chromium (Cr), Cr(VI) is the most toxic, with a toxicity 100 times higher than that of Cr(III) [1]. It has been reported that 0.1 mg L^−1^ of Cr(VI) is the threshold for aquatic organisms to stay alive [2]. Moreover, chromium in soil can affect crop metabolism and nutrient uptake. The Cr(VI) accumulation in the human body through the food chain harms internal organs, induces gene mutations, and causes cancer. Therefore, effective methods are needed to remove Cr(VI) in wastewater to protect the aquatic environment and human health.

Various methods, such as adsorption [3], chemical precipitation [4], ion exchange [5], and electrochemical techniques [6] have been applied to remove heavy metals. In addition, biological methods such as algae and microorganisms are also commonly used to remove hexavalent chromium from water [7,8]. Among these methods, adsorption is the most frequently adopted due to its simple operation, high efficiency, and low cost. Chen et al. prepared magnetic modified Enteromorpha prolifera biochar with a maximum adsorption capacity of 88.17 mg g^−1^ for Cr(VI) [2]. Sharma et al. prepared biochar with agricultural waste (Cornulaca-monacantha stem) as raw material, and immersed biochar in the mixture of NaOH (sodium hydroxide, 6%) and NaClO (sodium hypochlorite, 5.6%) to prepare modified biochar. The results showed that the maximum adsorption capacity of biochar and modified biochar for Cr(VI) were 37.17 mg g^−1^ and 67.88 mg g^−1^, respectively [9]. Many adsorbents, such as biochar [10], metal oxide nanoparticles [11], and graphene [12] have been explored for heavy metal removal. Biochar is considered as a promising adsorbent for its eco-friendly recycling. However, biochar has a limited capacity to remove heavy metal ions due to its small specific surface area and few functional groups [3,10]. Therefore, various strategies have been applied to modify biochar to improve its adsorption performances.

Chemical modification, including acid/base treatment [13], chemical oxidation [3], and impregnation with mineral oxides [14], is one of the most effective approaches to functionalize biochar. For example, biochar functionalized with KMnO_4_ as a strong oxidizing agent exhibited a high adsorption capacity of organic/inorganic pollutants due to the increased complexation effect of oxygen-containing functional groups [15]. Moreover, biochar modified with aluminum chloride (AlCl_3_) had an improved adsorption capacity of As(V) [16]. While many methods focused on the improvement of adsorption capacity via single modification, the impact of dual-modifiers on the removal performance of biochar still needs to be explored.

The removal of Cr(VI) from aqueous solution by bimetallic oxide-modified biochar has thus far been rarely studied. Here, a dual-modification strategy to prepare a novel biochar to remove Cr(VI) in solution is reported. The prepared bimetallic oxide modified biochar may have a large specific surface area and rich functional groups (MOx), which will have better Cr(VI) removal ability. The formation mechanism of biochar and its Cr(VI) removal mechanism were also investigated. The recycling of forest waste resources has been realized, and the technology of preparing biochar with dual-modifiers has been enriched in the academic circle. Specifically, the biochar (BC), prepared from chinar (Platanus orientalis Linn.) leaves, was first activated with K_2_CO_3_. Then the dual modifiers, KMnO_4_ and AlCl_3_, were embedded on the pre-activated biochar (KBC) by one-time modification. As a result, the dual-modified biochar (AMKBC) showed a significant improvement of physico-chemical properties and Cr(VI) removal capacity compared to the single-modified biochar.

## 2. Materials and Methods

### 2.1. Synthesis of Biochar

As a kind of forestry waste, the carbon content of chinar leaves is high, which has great advantages in preparing biochar. The high-value utilization of chinar leaves can also reduce the pollution caused by incineration. Leaves of chinar were collected on the campus of Qilu University of Technology (Jinan, China). A certain amount of crushed leaves was pyrolyzed in a tube furnace (Jinan Kester Experimental Equipment Co., Ltd., Jinan, China) at 800 °C for 2 h (BC). Crushed leaves mixed with K_2_CO_3_ (Tianjin Hengxing Chemical Reagent Manufacturing Co., Ltd., Tianjin, China) at a mass ratio of 50% was similarly pyrolyzed to make KBC. For the preparation of KMnO_4_ (Yantai Yuandong Fine Chemicals Co., Ltd., Yantai, China)/AlCl_3_·6H_2_O (Sinopharm Chemical Reagent Co., Ltd., Shanghai, China) modified biochar composites, KBC (0.25, 0.5, 1, and 1.5 g) was added into a 4 mL mixed solution of AlCl_3_·6H_2_O (0.6953 g) and KMnO_4_ (0.0759 g). The mixtures were dried and then pyrolyzed at 600 °C for 1 h to obtain AMKBC. Based on the mass ratio of KMnO_4_/AlCl_3_·6H_2_O to the added KBC, the KMnO_4_/AlCl_3_·6H_2_O modified KBC composites were named AMKBC_3/1_, AMKBC_3/2_, AMKBC_3/4_, and AMKBC_1/2_.

### 2.2. Materials Characterization

A scanning electron microscope (SEM) (JSM-7610F, JEOL, Akishima, Japan) combined with energy dispersive spectrometer (EDS) (X-max, Oxford, UK) was used to analyze the surface morphology and elemental composition of various biochars. The specific surface area (SSA) and pore-size distribution were assayed by N_2_ adsorption–desorption tests using a Brunner–Emmet–Teller (BET) surface area and pore size analyzer (BK300C, Gao Bo Science and Technology Co., Ltd., Beijing, China). The crystalline phases were obtained by X-ray diffraction analysis (XRD) (D8-ADVANCE, Bruker AXS, Karlsruhe, Germany). The chemical states and elemental composition of the tested metal oxides were assessed using X-ray photoelectron spectroscopy (XPS) (ESCALAB Xi+, ThermoFisher, Brno, Czech Republic).

### 2.3. Adsorption Measurements

To six identical 30 mL Cr(VI) solutions (100 mg L^−^^1^), 0.01 g of BC, KBC, AMKBC_3/1_, AMKBC_3/2_, AMKBC_3/4_, or AMKBC_1/2_ were added. The Cr(VI) concentration after 5 h shaking was measured according to our previously published method [5]. Three replicates were set for the adsorption experiments, and statistical analyses and least significant difference test for mean comparisons were conducted in SPSS (17.0, IBM company, Stanford, CA, USA). Differences at *p* ≤ 0.05 were considered significant. The adsorption capacity (*q*) of these materials was calculated (Equation (1)) [5].

(1) $${Q\;=\;(C}_{0}\;-\;C)V/m$$

where *C*_0_ (mg/L) and *C* (mg/L) represent the concentration of Cr(VI) before and after adsorption, respectively, *V* (L) is the volume of experimental solution, and *m* (g) is the mass of the adsorbent.

#### 2.3.1. Effect of pH on the Removal of Cr(VI) in Solution

The optimal biochar (0.01 g) was added into Cr(VI) solution (100 mg L^−1^), and the pH value of the mixture was adjusted to 3, 5, 7, or 9. The Cr(VI) removal capacity from the solution was measured according to our previously published method after shaking for 13 h [5].

#### 2.3.2. Adsorption Kinetic and Isothermal Adsorption Experiments

The adsorption kinetic (Equations (2)–(4)) and isothermal adsorption (Equations (5) and (6)) experiments were performed in this study according to our previous method [5]. The experimental parameters of adsorption kinetic were as follows: AMKBC_3/4_ dosage was 0.67 g L^−^^1^, the effect of contact time was 0–13 h, and solution concentration was 100 mg L^−1^ (pH = 3). The concentration of solution was changed (50–200 mg L^−1^) for the isothermal adsorption experiment, with the other parameters having been chosen because they were the best parameters obtained in the above experiment.

(2) $${log(q}_{e}{\;-\;q}_{t}{)\;=\;logq}_{e}{\;-\;(K}_{1}/2.303)\;\cdot \;t$$

(3) $$t/q_{t}\;=\;1/{(K}_{2}{\;\cdot \;q}_{e}^{2})\;+\;(1/q_{e})\;\cdot \;t\;$$

(4) $$q_{t}{\;=\;K}_{\mathrm{id}}{\;\cdot \;t}^{1/2}\;+\;C$$

(5) $$q_{e}{\;=\;q}_{m}K_{L}C_{e}{/(1\;+\;K}_{L}C_{e})\;$$

(6) $$q_{e}{\;=\;K}_{F\;}{\;\cdot \;C}_{e}^{1/n}\;$$

where *q_t_* (mg g^−1^) and *q_e_* (mg g^−1^) represent the adsorption amounts at time *t* (h) and equilibrium state during the adsorption process, respectively. *K*_1_ (h^−1^) and *K*_2_ (g mg^−1^ h^−1^) are the rate constants for the pseudo-first order and pseudo-second order adsorption kinetics. *K_id_* (mg g^−1^ min^−1/2^) is the intra-particle diffusion rate constant, and *C* is a constant related to the thickness of the boundary layer. Coefficient *q_m_* (mg g^−1^) is the maximum adsorption capacity, *C_e_* (mg L^−1^) is the equilibrium concentration of solution, and *n* is empirical index. Coefficients *K_L_* (L mg^−1^) and *K_F_* are indicators of adsorption capacity for Langmuir and Freundlich models.

### 2.4. Computational Method

The MnO (001)-terminated and Al_2_O_3_ (010)-terminated surfaces were chosen as computational models based on 2 × 2 supercell and 2 × 3 supercell, respectively. The constructed supercells were isolated with a 15 Å vacuum space in the c direction. The computational calculations were performed with the QUANTUM-ESPRESSO code [17], which is based on the density functional theory (DFT) [18], with the Hubbard model (DFT + U) [19]. The effective U value of 3.9 eV was applied for Mn (3d). The electronic wave function was expressed by the combination of plane wave basis, and the generalized gradient approximation (GGA) method with the Perdew–Burke–Ernzerhof (PBE) functional was employed for exchange and correlation interactions [20]. The Brillouin zones were performed by the Monkhorst-Pack scheme sampled into 3 × 3 × 1 for both MnO and Al_2_O_3_.

The adsorption energy (*E_ads_* (eV)) for HCrO_4_^−^ and Cr_2_O_7_^2−^ on Al_2_O_3_ and MnO was estimated based on the following equation (Equation (7)) [17,18,19,20]:

*E_ads_* = *E*_*MOx*+*Cr*_ − (*E_MOx_* + *E_Cr_*) (7)

where *E_MOx+Cr_* (eV) and *E_MOx_* (eV), respectively, represent the total energy of the biochar with and without adsorbed Cr(VI), *E_Cr_* (eV) denotes the energy of an isolated Cr(VI). MOx stands for Al_2_O_3_ or MnO. By definition, *E_ads_* < 0 corresponds to favorable or exothermic adsorption of Cr(VI) on MOx.

## 3. Results

### 3.1. AMKBC_3/4_ Greatly Decreased Cr(VI) Content in Solution

**Figure 1:**
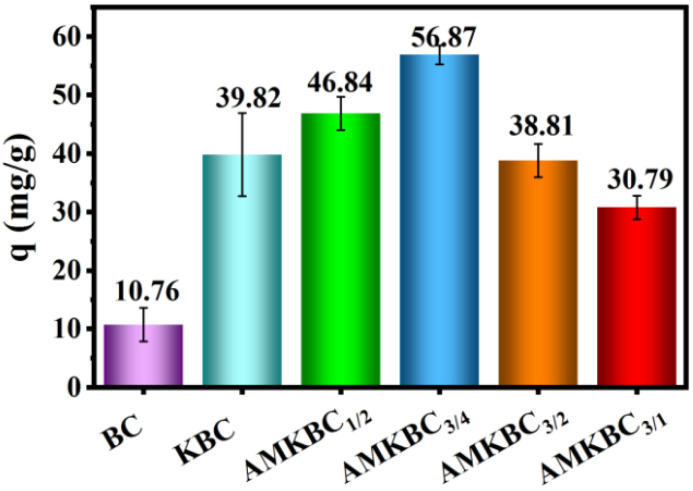
Cr(VI) adsorption capacity of various biochars: initial pH; T = 5 h; m = 0.01 g; V = 30 mL; C_0_ = 100 mg L^−1^.

The Cr(VI) adsorption capacity of BC and KBC were 10.76 mg g^−1^ and 39.82 mg g^−1^ (Figure 1), respectively. Notably, the adsorption capacity of AMKBC had a bell curve as the mass ratio of KMnO_4_/AlCl_3_·6H_2_O to KBC increased (Figure 1). Among all the samples, AMKBC_3/4_ exhibited the highest adsorption capacity of 56.87 mg g^−1^. Compared with the maximum adsorption capacity (38 mg g^−1^) of Mg/Al modified biochar for Cr(VI) in the literature [1], the biochar prepared in this study has better adsorption performance. The further decrease of Cr(VI) adsorption capacity was attributed to the blocking effect of Al/Mn metal oxides on the pores of the biochar.

### 3.2. Characterization of BC, KBC, and AMKBC_3/4_

**Figure 2:**
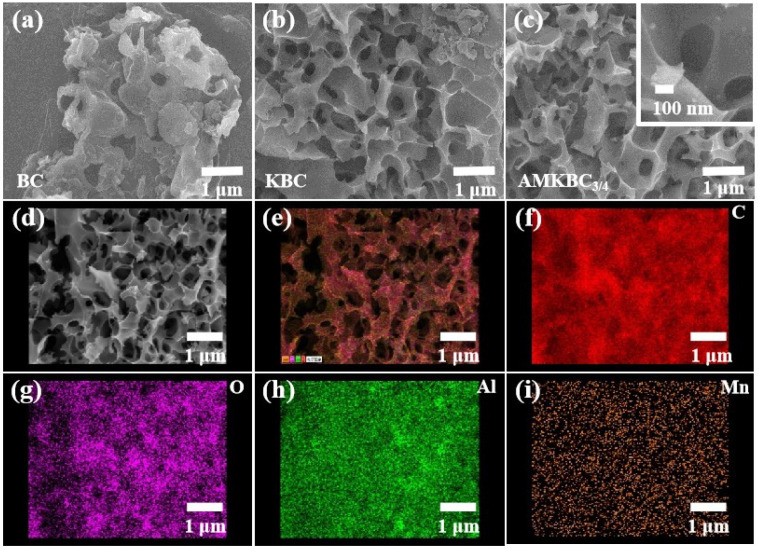
SEM images of BC (**a**), KBC (**b**) and AMKBC_3/4_ (**c**,**d**); EDS merged image of total elements (**e**); the distribution maps of C (**f**), O (**g**), Al (**h**), and Mn (**i**) for AMKBC_3/4_.

In the SEM images, BC had a small number of pores (Figure 2a), while KBC had a superior pore structure (Figure 2b). The gases (CO and CO_2_) produced from the chemical reaction between K_2_CO_3_ and carbon advantageously formed additional pores on the biochar surface except for the oxidation of carbon, which resulted in an increased interlayer distance [5].

AMKBC_3/4_ with porous structure was loaded with spherical particles, which might be Al/Mn oxides (Figure 2c, [21]). Similarly, KBC with a large surface area could be the carrier for nano Al/Mn oxides to reduce agglomeration, which facilitated a high adsorption capacity of the pollutants [22]. The EDS results indicated that Al and Mn oxides had loaded on the surface of AMKBC_3/4_ (Table S1), which matched well with the results from elemental distribution diagrams (Figure 2d–i). The presence of C and O indicated the existence of rich oxygen-containing functional groups on the surface of AMKBC_3/4_ (Figure 2f,g). As shown in Figure 2e, the uniform display of each element on the surface of AMKBC_3/4_ indicates that Al/Mn oxides did not agglomerate, which would have provided additional adsorption sites for Cr(VI) removal.

**Figure 3:**
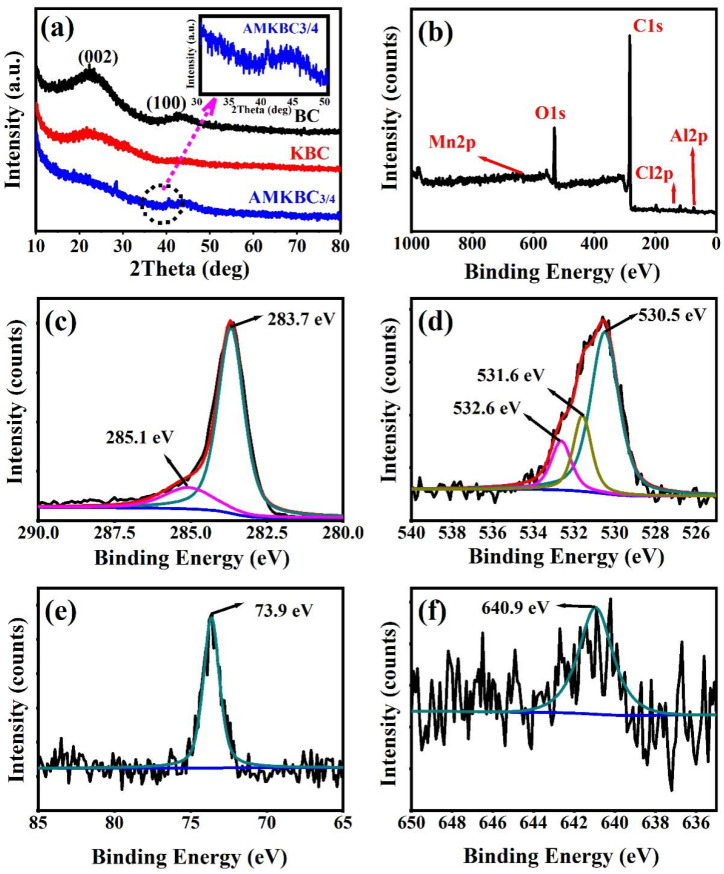
X-ray diffraction analysis of BC, KBC, and AMKBC_3/4_ (**a**). For AMKBC_3/4_: XPS spectra (**b**); high resolution XPS spectra of C1s (**c**); O1s (**d**); Al2p (**e**); Mn2p3/2 (**f**).

In Figure 3a, the graphite crystal plane diffraction peaks ((002) and (100)) were measured to be 23° and 43°, respectively, for both BC and KBC [23]. Notably, the remarkable diffraction peaks of Al_2_O_3_ appeared at 18.71° (JCPDS: 88–1609) and 28.42° (JCPDS: 88–0107), and the peak situated at 40.49° is assigned to MnO [24]. Previous studies have found that the existence of metal oxides on the surface of biochar could enhance the removal capacity of anions via coordination reaction [25]. The loaded Al/Mn oxides on the biochar surface can react with HCrO_4_^−^ and Cr_2_O_7_^2−^, resulting in effective Cr(VI) removal [26].

The visible peaks of C1s, O1s, Al2p, and Mn2p for AMKBC_3/4_ (Figure 3b–f) confirmed the loading effect of Al/Mn oxides, which was consistent with the EDS analysis. The peaks located at 283.7 eV and 285.1 eV represented the functional groups of C–N and C=C of the AMKBC_3/4_ in the high-resolution XPS spectrum (Figure 3c) [27]. The O1s spectrum had three peaks (Figure 3d): the peak of 531.6 eV can be ascribed to the lattice oxygen (CO, C=O) [28]; the peaks of 532.6 eV and 530.5 eV are attributed to the oxygen from organic matter (–OH or C–O–C) [29] and inorganic matter (MO and M–OH, M was metal), respectively [28]. The spectrum of Al2p and Mn2p3/2 represented elemental aluminum and manganese from Al_2_O_3_ and MnO (Figure 3e,f), respectively [30]. The loaded Al/Mn oxides with different chemical states on the surface of AMKBC_3/4_ could react with Cr_2_O_7_^2−^ and HCrO_4_^−^ through a complex reaction and electrostatic attraction, which enhanced Cr(VI) removal (Equations (8) and (9)). A similar result was obtained by Wu, who found that Al/Mn oxides could form complexes with As(III) and As(V) [4].

(8) R–O–M + Cr(VI) ↔ R–O–M⋯Cr(VI)

(9) R–M–OH + Cr(VI) ↔ R–M–OH⋯Cr(VI)

**Figure 4:**
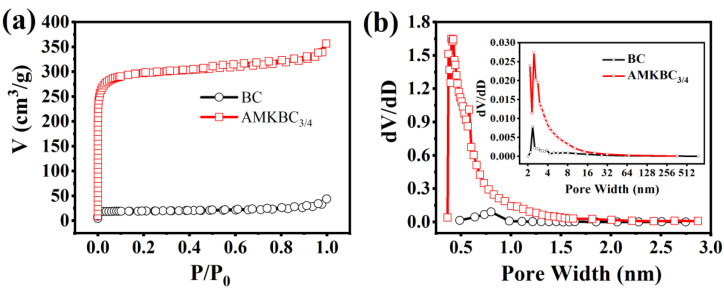
N_2_ adsorption-desorption isotherms (**a**), and pore size distribution (**b**) of BC and AMKBC_3/4_.

AMKBC_3/4_ had a higher percentage of micropores than BC when the relative pressure (P/P_0_) < 0.1 (Figure 4a). The hysteresis loop indicated that both BC and AMKBC_3/4_ had mesoporous structure (Figure 4a, [31]). Moreover, the upward curve indicated the existence of macropores for these biochars when the relative pressure (P/P_0_) was close to 1.0 (Figure 4a, [32]). A similar result was obtained from the pore volume distribution (Figure 4b). Accordingly, the specific surface area and the total pore volume of AMKBC_3/4_ were 1173.36 m^2^ g^−1^ and 0.54 cm^3^ g^−1^, respectively (Table 1), which was 15.25 and 8.87 times higher than that of biochar. The surface area and volume of micropore reached 93.97% and 83.92%, which was significantly higher than those of BC (85.37% and 47.54%). The increased surface area and pore volume of AMKBC_3/4_ might be attributed to the large surface area of loaded Al/Mn oxides and gasification of K_2_CO_3_ [5], which enhanced Cr(VI) removal.

### 3.3. Effect of pH on the Removal of Cr(VI) in Solution

**Figure 5:**
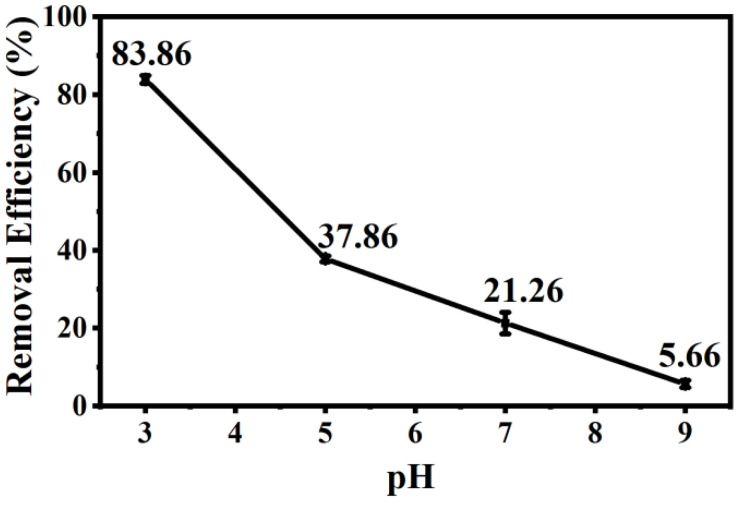
Effect of pH on the removal of Cr(VI) in solution: pH = 3–9; T = 13 h; m = 0.01 g; V = 15 mL; C_0_ = 100 mg L^−1^.

**Figure 6:**
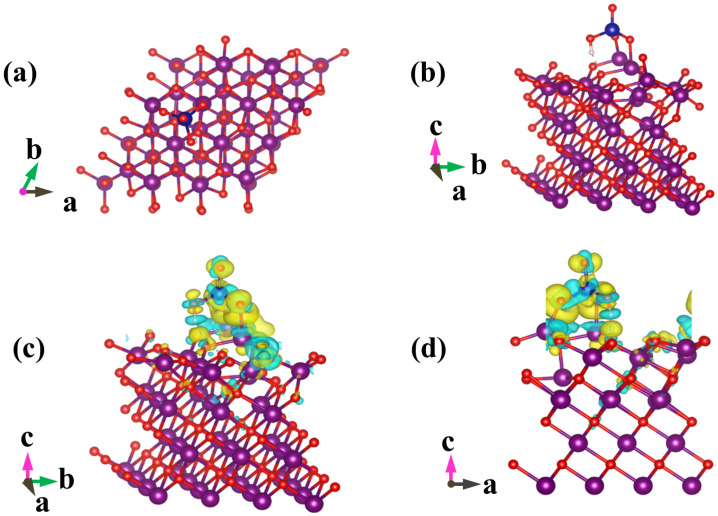
Optimized geometric configurations for HCrO_4_^−^ adsorbed on MnO(001) (**a**,**b**), and isosurface representations of charge difference (**c**,**d**). The accumulation and depletion of electrons are represented by the yellow and cyan regions, respectively. Isosurface value = 0.002 e/bohr^3^. Manganese atoms are purple, oxygen atoms are red, chromium atoms are blue, and hydrogen atoms are white.

**Figure 7:**
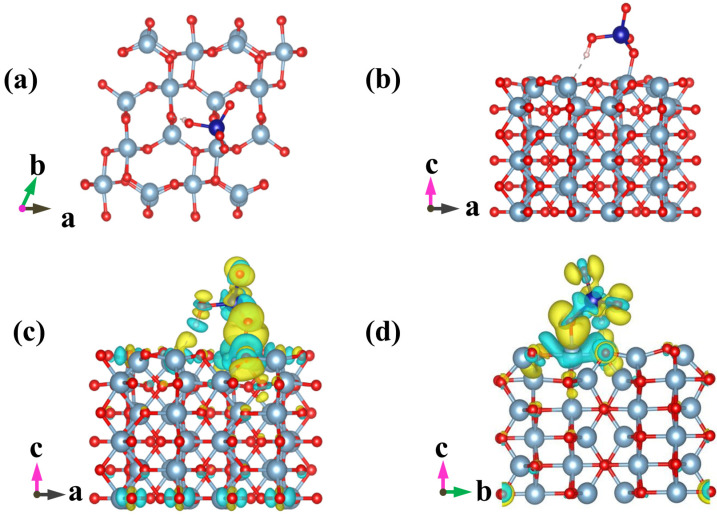
Optimized geometric configurations for HCrO_4_^−^ adsorbed on Al_2_O_3_(010) (**a**,**b**), and isosurface representations of charge difference (**c**,**d**). The accumulation and depletion of electrons are represented by the yellow and cyan regions, respectively. Isosurface value = 0.002 e/bohr^3^. Aluminum atoms are gray, oxygen atoms are red, chromium atoms are blue, and hydrogen atoms are white.

Chromium mainly exists in the form of HCrO_4_^−^ and Cr_2_O_7_^2−^ under acidic conditions; removal efficiency reached 83.86% when the pH value of solution was 3 (Figure 5), which could be ascribed to the following three favorable factors: (i) low adsorption energy for HCrO_4_^−^ and Cr_2_O_7_^2−^ according to DFT calculations, as shown in Figure 6 and Figure 7 and Table S2 [14]; (ii) high redox potential of Cr(VI) promoting the reduction of Cr(VI) to Cr(III) at low pH conditions [29]; (iii) enhanced electrostatic attraction due to the increased zeta potential of AMKBC_3/4_ while in the form of CrO_4_^2−^ under alkaline conditions [5]. The removal rate of Cr(VI) is due to the Al/Mn oxides dopant [1]. Conversely, the decreased Cr(VI) removal rate with the increased pH was because OH– and CrO_4_^2−^ ions competed for unfilled adsorption sites of AMKBC_3/4_ [14]. Based on the above analysis, the optimum pH of the solution and the corresponding optimal dosage of AMKBC_3/4_ were set to 3 and 0.67 g L^−1^, respectively, in the subsequent experiments to obtain excellent adsorption performance.

### 3.4. Adsorption Kinetics

The influence of contact time on the Cr(VI) adsorption is presented in Figure S1a. Specifically, the Cr(VI) adsorption capacity of AMKBC_3/4_ increased quickly in the first hour, and then it slowly increased until the adsorption achieved equilibrium of 125.66 mg g^−1^ at the 8th hour. The rapid Cr(VI) adsorption was due to the large amount of effective adsorption sites and functional groups on the surface of AMKBC_3/4_. Likewise, the reduced Cr(VI) adsorption rate was due to the reduced adsorption sites and the increased diffusion resistance with the extension of the adsorption process.

The Cr(VI) diffusion to the AMKBC_3/4_ surface according to Weber–Morris intraparticle diffusion modeling is presented in Figure S1b, and the external surface adsorption and the intraparticle diffusion along the pores of the AMKBC_3/4_ occurred before the Cr(VI) adsorption achieved equilibrium. A similar result was reported by our previous study as well [5]. The pseudo-first order model and the pseudo-second order model reflected the Cr(VI) adsorption process of AMKBC_3/4_ (Figure S1c,d): the correlation coefficient of pseudo-second order model was higher than that of pseudo-first order model, indicating that the Cr(VI) adsorption on AMKBC_3/4_ was mainly chemical adsorption. Moreover, the theoretical value of the adsorbed Cr(VI) according to the pseudo-second order model was 126.58 mg g^−1^, which was close to the experimental value of 125.66 mg g^−1^.

### 3.5. Adsorption Isotherms

The isothermal Cr(VI) adsorption models on AMKBC_3/4_ is shown in Figure S2. The correlation coefficient of the Langmuir model was higher than that of the Freundlich model, indicating that the monolayer Cr(VI) adsorption occurred on AMKBC_3/4_. Meanwhile, the calculated Cr(VI) adsorption capacity on AMKBC_3/4_ from the Langmuir model was 158.73 mg g^−1^, which was close to the maximum adsorption capacity of 152.86 mg g^−1^. Compared with the modified biochar (67.88 mg g^−1^) prepared by soaking biochar in the mixture of NaOH and NaClO by Sharma et al. [9], the biochar prepared in this study has a larger adsorption capacity for Cr(VI), and the doping of metal oxides plays a significant role in this process. The Cr(VI) adsorption capacity by various modifiers-produced biochars is compared in Table 2, which indicates that the AMKBC_3/4_ has a superior Cr(VI) adsorption capacity. Compared with single modification, the biochar prepared in this study has a higher adsorption capacity, which proves that the dual modification can indeed enhance the adsorption capacity of biochar compared to the single modification. The following reasons could explain the Cr(VI) removal in the solution: chromate ions implanted the pores of the AMKBC_3/4_ due to its surface structure destruction from the release of Al^3+^/Mn^4+^ [33], and electrostatic attraction occurred between chromate ions and reattached Al^3+^/Mn^4+^ by AMKBC_3/4_ (Equation (10)). The existence of metal oxides on the surface of AMKBC_3/4_ could enhance the removal capacity of HCrO_4_^−^ and Cr_2_O_7_^2−^ via coordination reaction [25].

(10) $${R–M}^{n+}{\;+\;\mathrm{Cr}}^{6+}\;↔{\;R–M}^{n+}{\cdots \mathrm{Cr}}^{6+}$$

### 3.6. Removal Mechanism of Cr(VI) in Solution

**Figure 8:**
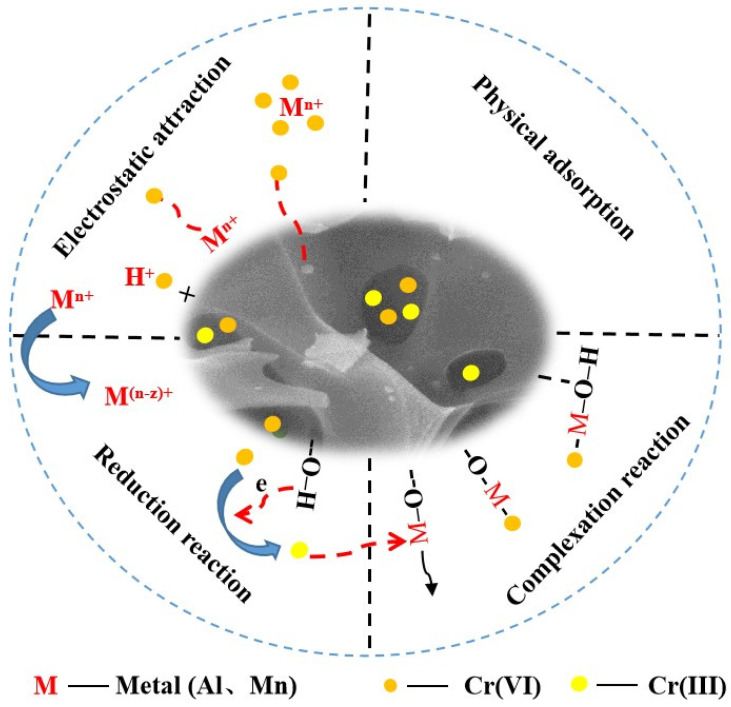
Cr(VI) removal mechanism of AMKBC_3/4_.

While the KBC served as the carrier for Al/Mn oxides with reduced agglomeration, the loaded Al/Mn oxides could also enhance the adsorbent surface area. Therefore, the Cr(VI) removal by AMKBC_3/4_ in solution was dependent on its pore structure and on the loaded Al/Mn oxides. Overall, the possible approaches for Cr(VI) removal by AMKBC_3/4_ were summarized (Figure 8): (i) the functional groups and loaded metal cations from protonated adsorbent could adsorb HCrO_4_^−^, Cr_2_O_7_^2−^, and CrO_4_^2−^ via electrostatic attraction [38]; (ii) Cr(VI) could be reduced to Cr(III) by hydroxyl group at a lower pH (Equation (11)) [39], followed by the Cr(III) chelation with the Al/Mn oxides on the surface of AMKBC_3/4_ (Equations (12)–(14)); (iii) Cr(VI) could implant the pores of AMKBC_3/4_.

(11) $${3R–\mathrm{OH}\;+\;\mathrm{HCrO}}_{4}{}^{-}\;{+\;4H}^{+}\;↔\;{3R–O\;+\;\mathrm{Cr}}^{3+}\;{+\;4H}_{2}O$$

(12) $${R–O–M+\mathrm{Cr}}^{3+}\;↔{\;R–O–M\cdots \mathrm{Cr}}^{3+}$$

(13) $${R–M–\mathrm{OH}+\mathrm{Cr}}^{3+}\;↔{\;R–M–\mathrm{OH}\cdots \mathrm{Cr}}^{3+}$$

(14) $${R–\mathrm{MOOH}+\mathrm{Cr}}^{3+}\;↔{\;R–\mathrm{MOOH}\cdots \mathrm{Cr}}^{3+}$$

## 4. Techno Economic Challenges and Future Research Directions

The technological challenge to preparing dual-modified biochar is to find an appropriate ratio of KBC and Al/Mn oxides in order to make metal oxides that can be evenly distributed on the biochar surface and do not block the biochar channels. In the future, dual-modified biochar can be applied to treat organic wastewater, and the removal mechanism can also be explored. Moreover, we can try to apply dual-modified biochar to treat natural water bodies.

## 5. Conclusions

In summary, a novel biochar has been developed as an adsorbent for removal of Cr(VI) in solution with a dual-step strategy. When the dosage of KBC was 1 g, AlCl_3_·6H_2_O is 0.6953 g, and KMnO_4_ was 0.0759 g, the modified biochar had the best adsorption performance, and had a maximum Cr(VI) saturated adsorption capacity of 152.86 mg g^−1^. The feasibility of dual-modified biochar was verified, which will provide reference for the preparation process and formation mechanism of other dual-modified biochar. The modified biochar of AMKBC_3/4_ demonstrated its potential for removing Cr(VI) due to the synthetic effect of electrostatic attraction, reduction reaction, complexation reaction and physical adsorption in solution. The spontaneous adsorption process agreed well with the pseudo-second order and Langmuir models. Overall, this present study provided not only a novel modification route for biochar, but also possibilities to remediate water polluted with other heavy metals.

## Figures and Tables

**Table 1:** The textural parameters of BC and AMKBC_3/4_.

Adsorbent	S_BET_ (m^2^ g^−1^)	V_tot_ (cm^3^ g^−1^)
BC	76.96	0.06
AMKBC_3/4_	1173.36	0.54

**Table 2:** Adsorption capacities of Cr(VI) by modified biochars derived from various feedstocks.

Adsorbent	Modifier	Cr(VI) Concentration	pH	Adsorption Capacity (mg g^−1^)	Reference
Enteromorpha prolifera	FeCl_3_	100 mg L^−1^	5.03	88.17	[2]
Jute fibers	H_3_PO_4_	10–100 mg L^−1^	2–3	77.34	[13]
Jute fibers	KOH	10–100 mg L^−1^	2–3	42.00	[13]
Water hyacinth	Nano-ZnO	25–300 mg L^−1^	natural	43.48	[34]
Sludge	Nanoscale zero-valent iron	100 mg L^−1^	4	64.13	[35]
Cron straw	H_3_PO_4_	60–1050 mg L^−1^	7	116.28	[36]
activated carbon	AlCl_3_	5–65 mg L^−1^	5.38	33.74	[37]
activated carbon	MnCl_2_	5–65 mg L^−1^	5.38	33.67	[37]
Chinar leaves	K_2_CO_3_^−^ KMnO_4_/AlCl_3_·6H_2_O	50–200 mg L^−1^	3	152.86	In this study

## Data Availability

The data presented in this study are available on request from the corresponding author.

## Supplementary Materials

The following are available online at https://www.mdpi.com/article/10.3390/polym14010039/s1, Figure S1: Effect of contact time on the removal of Cr(VI) (a), Weber-Morris intra-particle diffusion model (b), pseudo-first order model (c) and pseudo-second order model (d) for Cr(VI) adsorption of AMKBC_3/4_. (pH = 3, T = 0–13 h, m = 0.01 g, V = 15 mL, C0 = 100 mg L^−^^1^), Figure S2: Langmuir isotherm (a) and Freundlich isotherm (b) for Cr(VI) adsorption of AMKBC_3/4_. (pH = 3, T = 13 h, m = 0.01 g, V = 15 mL, C0 = 50–200 mg L^−1^), Table S1: Elemental content of adsorbents, Table S2: DFT calculated adsorption energy (Eads, eV) of HCrO_4_^−^ and Cr_2_O_7_^2−^ for the favored adsorption configurations on MnO (001) and Al_2_O_3_ (010).
