# Supplementary material for: Chemically Dual-Modified Biochar for the Effective Removal of Cr(VI) in Solution
Source: Polymers (Basel). 2021 Dec 23;14(1):39. doi: 10.3390/polym14010039 (PMC8747338; doi:10.3390/polym14010039)
Supplement: Supplementary file 1 [file polymers-14-00039-s001.zip › polymers-1491981-supplementary.pdf]

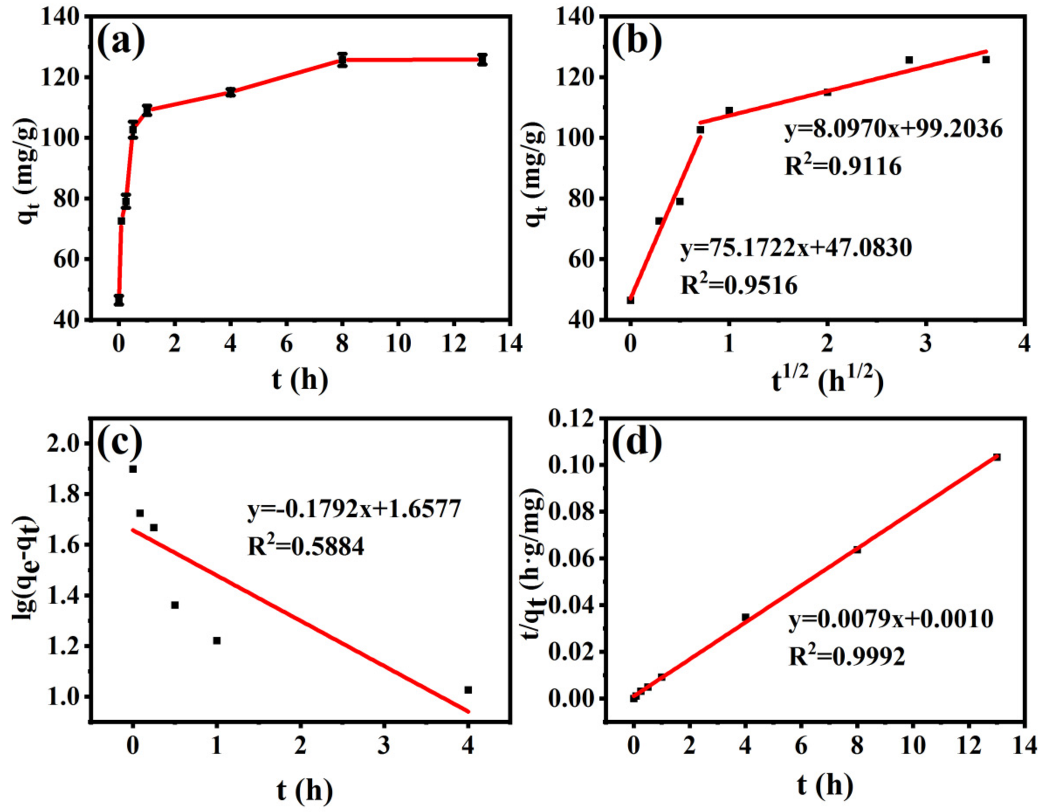

Figure S1. Effect of contact time on the removal of Cr(VI) (a), Weber-Morris intra-particle diffusion model (b), pseudo-first order model (c) and pseudo-second order model (d) for Cr(VI) adsorption of $AMKBC_{3/4}$. (pH=3, T=0-13 h, m=0.01 g, V=15 mL, C_0=100 mg L^{-1})

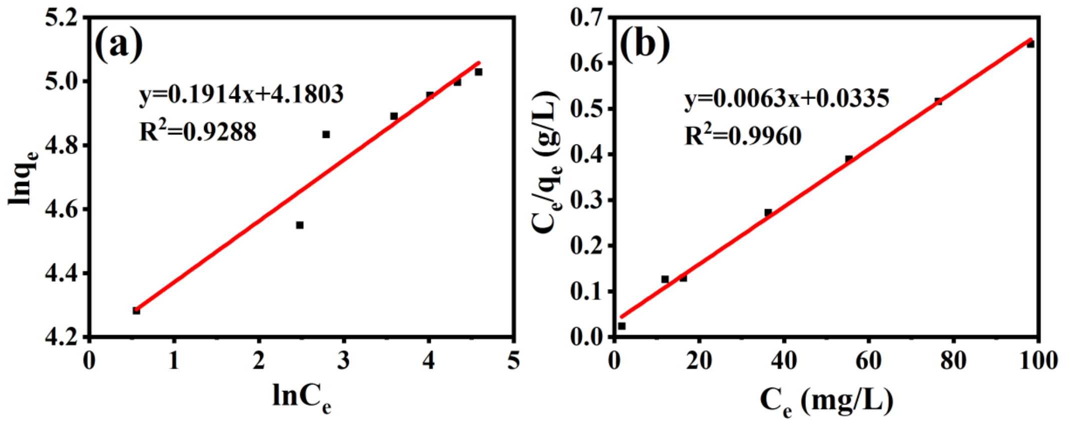

Figure S2. Langmuir isotherm (a) and Freundlich isotherm (b) for Cr(VI) adsorption of $AMKBC_{3/4}$. (pH=3, T=13 h, m=0.01 g, V=15 mL, C_0=50-200 mg L^{-1})

Table S1 Elemental content of adsorbents

Adsorbent	C(wt%)	O(wt%)	Al(wt%)	Mn(wt%)
BC	86.4	13.6	/	/
KBC	92.6	7.4	/	/
$AMKBC_{3/4}$	83.3	11.5	4.5	0.7

Table S2. DFT calculated adsorption energy (Eads, eV) of $HCrO_4^-$ and $Cr_2O_7^{2-}$ for the favored adsorption configurations on MnO (001) and Al_2O_3 (010).

-	$HCrO_4^-$	$Cr_2O_7^{2-}$
MnO(001)	-0.52	-0.21
Al_2O_3(010)	-1.64	-2.01
